# Testosterone, Plumage Colouration and Extra-Pair Paternity in Male North-American Barn Swallows

**DOI:** 10.1371/journal.pone.0023288

**Published:** 2011-08-10

**Authors:** Cas Eikenaar, Megan Whitham, Jan Komdeur, Marco van der Velde, Ignacio T. Moore

**Affiliations:** 1 Department of Biological Sciences, Virginia Tech, Blacksburg, Virginia, United States of America; 2 Behavioural Ecology and Self-Organisation, Centre for Ecological and Evolutionary Studies, University of Groningen, Haren, The Netherlands; 3 Animal Ecology Group, Centre for Ecological and Evolutionary Studies, University of Groningen, Haren, The Netherlands; Arizona State University, United States of America

## Abstract

In most monogamous bird species, circulating testosterone concentration in males is elevated around the social female's fertile period. Variation in elevated testosterone concentrations among males may have a considerable impact on fitness. For example, testosterone implants enhance behaviours important for social and extra-pair mate choice. However, little is known about the relationship between natural male testosterone concentration and sexual selection. To investigate this relationship we measured testosterone concentration and sexual signals (ventral plumage colour and tail length), and determined within and extra-pair fertilization success in male North American barn swallows (*Hirundo rustica erythrogaster*). Dark rusty coloured males had higher testosterone concentrations than drab males. Extra-pair paternity was common (42% and 31% of young in 2009 and 2010, respectively), but neither within- nor extra-pair fertilization success was related to male testosterone concentration. Dark rusty males were less often cuckolded, but did not have higher extra-pair or total fertilization success than drab males. Tail length did not affect within- or extra-pair fertilization success. Our findings suggest that, in North American barn swallows, male testosterone concentration does not play a significant direct role in female mate choice and sexual selection. Possibly plumage colour co-varies with a male behavioural trait, such as aggressiveness, that reduces the chance of cuckoldry. This could also explain why dark males have higher testosterone concentrations than drab males.

## Introduction

In most seasonally breeding socially monogamous birds in which males care for young, male circulating testosterone concentration increases rapidly at the beginning of the breeding season to reach a breeding baseline level [1]. Within a breeding cycle male circulating testosterone concentration rises above the breeding baseline level around the fertile period of the social female (hereafter referred to as elevated testosterone concentration) [1]. Although this pattern of testosterone secretion is typical for all reproductively active males in a population, considerable variation exists among males in elevated testosterone concentrations [2]–[4]. This variation may have a significant impact on individual fitness, as testosterone appears to have a prominent role in mediating sexually selected traits [5]–[10]. Observational studies suggest that mate guarding, a direct result of male-male competition over fertilizations, is correlated with high male testosterone concentrations around the fertile period of the social female [5]–[7], and cuckoldry risk is correlated with male testosterone concentrations around the fertile period of the social female [4]. Moreover, experimental studies in which male elevated testosterone concentrations were temporally extended have shown that testosterone enhances behaviours important for social and extra-pair female mate choice, such as singing [8], vigilance behaviour [9], and display frequency to receptive females [10].

Testosterone implants have also been shown to enhance sexually selected physical characters, such as crown ultraviolet chroma [11], and hence could influence a male's attractiveness as a (extra-pair) mate. Evidence for a role of testosterone in male extra-pair behaviour comes from two experimental field studies. In dark-eyed juncos (*Junco hyemalis*) testosterone implants resulted in lower cuckoldry rates and higher chances of gaining extra-pair fertilizations [12]. In contrast, blue tit males (*Cyanistes caeruleus*) with experimentally enhanced testosterone concentrations had higher paternity losses and gained no more extra-pair fertilizations than control males [13]. The contrasting findings of these two studies highlight the lack of understanding concerning the role of testosterone in extra-pair behaviour.

While we have only limited information on supplementary testosterone and extra-pair behaviour, equally little is known about the relationship between natural male elevated testosterone concentrations and individual reproductive success. Studies on several polygynous species revealed that male elevated testosterone concentration was positively correlated with copulation success (satin bowerbirds (*Ptilonorhynchus violaceus*) [14]; black grouse (*Tetrao tetrix*) [2]) and ultimately fledgling success (red-winged blackbirds (*Agelaius phoeniceus*) [15]). Similar to a polygynous mating system, extra-pair copulations have the potential to tremendously increase the variation in male reproductive success in socially monogamous species [16]. It is therefore surprising that there appears to be a lack of studies relating natural variation in male elevated testosterone concentrations with variation in reproductive success resulting from extra-pair behaviour. Another incentive to collect correlational data on testosterone and (extra-pair) mating success is that manipulative studies may lead to levels of testosterone that are too high to mimic the natural situation [17], [18], and can even lead to temporal male infertility [17].

We measured male elevated testosterone concentration and determined within and extra-pair fertilization success in North American barn swallows (*Hirundo rustica erythrogaster*). This is a suitable study species, as extra-pair paternity is common (e.g. 31% [19]) and apparently independent of male age, a potentially confounding factor in paternity studies [20]. More importantly, individual male elevated testosterone concentrations positively correlate with ventral plumage colouration, a trait involved in sexual selection; darker coloured males have higher annual apparent reproductive success [21], and experimental enhancement of male plumage colour reduced cuckoldry rates [22]. Our study is the first to correlate male endogenous testosterone concentration with reproductive success. Given the direction of the above relationships, we predicted that males with naturally high elevated testosterone concentrations face a lower risk of being cuckolded and have higher annual reproductive success than males with lower elevated testosterone concentrations.

## Methods

### Ethics statement

All procedures conformed to the guidelines outlined by the National Institutes of Health Guide for the Care and Use of Laboratory Animals and were also approved by the Virginia Tech Animal Care Committee (IACUC #08-258-BIOL).

### Study species

Barn swallows are small (ca 20 g) insectivorous semi-colonial passerines. North American barn swallows differ phenotypically from the European race (*H. r. rustica*) in having melanin-based rust-coloured feathers extending from the throat down the breast and belly to the vent [23]. Intra-specific brood parasitism (egg dumping) has been documented in European barn swallows [24], but appears to be absent in North American populations [25], [26].

### Study site and field procedures

The fieldwork in this study was carried out on the Virginia Tech campus (37°13′N, 80°25′W, elevation ca. 700 m) from April to August in the years 2009 and 2010. In 2009 we studied swallows breeding in a single barn. In 2010 we studied swallows breeding at the same barn site and at two additional barns, all positioned within a one-km radius from oneanother.

Adults were caught passively (without song playback, use of a dummy bird etc.) using mist nets around the fertile period of the females. All birds were caught from 0500–0700 hrs. They received a unique combination of two colour bands to allow visual identification at the nest. Length of the longest outermost tail feather (tail length) was measured to the nearest mm. A few feathers were plucked from the belly and mounted on an index card for later objective quantification of plumage colouration with an Ocean Optics reflectance spectrophotometer (see below). A blood sample (ca 150 µl) was taken from the wing vein within 10 minutes of capture. The plasma was separated within 4 hours of capture and frozen until hormone assaying. Birds were sexed by the presence (females) or absence (males) of a brood patch and by observations during incubation and provisioning of the nestlings. Molecular sexing confirmed our sexing in the field. CE performed all measurements.

In 2009 we banded, obtained a testosterone sample and analysed the parentage of the young in all the nests of 16 males. Four of these males were mated with a female that was also colour-banded. In 2010 we banded, obtained a testosterone sample and analysed the parentage of the young in all the nests of 35 males. Thirty-four of these males were mated with a female that was also colour-banded. Plumage colouration and parentage, but not testosterone, were quantified for two additional males. Three males and two females were caught and sampled at our study site in both years. Nests were inspected regularly to determine date of clutch initiation and clutch size. Visual observations during incubation and provisioning served to determine which birds belonged to which nests. A small (ca 10 µl) blood sample was taken from all nestlings a few days after hatching (n = 411), and a tissue sample was collected of embryos from eggs that failed to hatch (n = 13). Samples were stored in ethanol at room temperature for later parentage analyses.

### Plumage colour scoring

Each male's feather sample was scored three times using the reflectance spectrophotometer. We calculated three traditional indices of variation in colour - hue, brightness and saturation – and averaged these across the three replicate spectra. Hue was defined as the wavelength of maximum reflectance, brightness as the mean reflectance between 320–700 nm, and saturation as the sum of reflectance between 625–700 nm, divided by sum of reflectance between 320–700 nm [27]. Prior to calculations of hue, reflectance curves were smoothed by taking the running median over 5 nm intervals.

Principal components analysis was used to collapse hue, brightness and saturation scores into a single metric. The first principal component (PC1) explained 81% of the variation in the colour scores. Ventral feathers of more colourful, dark rusty males (with a high PC1 score) have higher hue (eigenvector = 0.54), are more saturated (eigenvector = 0.60) and less bright (eigenvector = −0.59) than those of drab males. In the data analyses we used this PC1 of colour and in the remainder of the text we refer to this measure as ‘ventral plumage colour’.

### Hormone analysis

All blood plasma samples from males were analyzed for testosterone in duplicate by radioimmunoassay following the procedures of [28], [29]. We performed a direct assay without chromatography, thus measuring total androgen (testosterone+5α-dihydrotestosterone). Sample volumes ranged from 20–69 µl (mean: 53 µl) and from 14–77 µl (mean: 55 µl) in 2009 and 2010, respectively. The limit of detection depends on plasma volumes and was ca 0.15 ng/ml in 2009 and ca. 0.14 ng/ml in 2010. The samples of each year were run in a single assay with a mean extraction efficiency of 84% and an intra-assay variation of 11.9% in 2009 and a mean extraction efficiency of 77% and an intra-assay variation of 12.8% in 2010. The inter-assay variation was 9.3%. The testosterone antibody used was T-3003s (Fitzgerald: Catalog # WLI-T3003s, New catalog #20R-TR018W). Typically in birds the concentration of circulating 5α-dihydrotestosterone is extremely low and often undetectable in individual blood samples (e.g. undetectable in 44% of 92 male barn swallow samples [3]), thus circulating testosterone concentrations will be extremely similar to circulating total androgen concentrations. We therefore hereafter refer to total androgen as testosterone.

### Molecular sexing and parentage analysis

DNA was extracted from blood and tissue samples using a chelex extraction method [30] or salt extraction method [31]. Sex of all DNA samples was determined following [32] and/or [33]. To exclude and assign paternity, parents and chicks were genotyped for four microsatellite loci: Esc6 [34], Pocc6 [35], Hru5 and Hru6 [36]. Estimates of variability and null-allele frequency of these microsatellite loci are presented in Table 1. PCR reactions were carried out in 10 µl volume using 20–50 ng of template DNA, a QIAGEN Multiplex PCR Kit and manufacturer's protocol. Final primer concentrations were 0.2 µM for Pocc6 and Hru5 and 0.1 µM for Esc6 and Hru6. Microsatellite loci Esc6 and Pocc6, and loci Hru5 and Hru6 were amplified simultaneously using the following PCR program: 15 min. At 95°C, 35 cycles of 94°C for 30 s, 50°C (Esc6, Pocc6) or 58°C (Hru5, Hru6) for 90 s and 72°C for 60 s, followed by 60°C for 30 min. Fluorescently labelled PCR products were separated on an AB3730 DNA analyzer. Subsequently, allele-lengths were determined using Genemapper 4.0 software. Using Cervus 3.0 [37], mean exclusion probability of the 4 markers was calculated to be 0.9915 for the first parent and 0.9989 when the identity of both social parents was known. Paternity of the social male was excluded if there were >1 mismatches (143 instances) between the genotypes of the social father and nestling, and those nestlings were regarded as extra pair young (EPY). Nestlings having one mismatch with their social father (4 instances) were regarded as within pair young unless another breeding male (extra-pair male) matched completely the genotype of such a nestling (2 instances). For the EPY we tried to assign paternity to one of the caught breeding males using Cervus 3.0 [37]. Extra pair males were assigned only when a male completely matched the offspring's genotype.

**Table 1:** Estimates of variability and null-allele frequency of 4 microsatellite loci in North American barn swallow.

Locus	A	He	F(null)
Hru5	20	0.91	+0.014
Hru6	58	0.97	−0.009
Esc6	16	0.85	−0.017
Pocc6	14	0.86	−0.010

A = number of alleles.

He = expected heterozygosity.

F(null) = estimated null allele frequency.

A, He, F(null) are estimated using Cervus 3.0.

### Data analyses

Individual testosterone concentration may vary as a result of differences in extrinsic factors at the time of sampling, such as sexual state (fertile or not) of the social mate [1], time of day [38] or breeding density [39]. Consequently, extrinsic factors could mask a potential relationship between male testosterone and fertilization success. To investigate the effect of various extrinsic factors on testosterone concentration, we performed a multilevel GLMM using MlwiN 2.0 [40]. As none of the interactions between any of the independent variables (extrinsic factors) and year were significant (all *p*>0.49), data of both years were lumped. The extrinsic factors entered into the model were: day of capture expressed as the number of days before or after the social mate's clutch initiation, time of capture, ambient temperature at capture, breeding density expressed as the number of active nests within 6 meters of the focal bird's nest, and opportunities for extra-pair activity expressed as the number of fertile females present per male in the barn on the day of capture. Breeding density was measured within 6 m, as this was the average distance males had to fly through the barns, and could thus interact with other swallows, to reach their nests. Extra-pair opportunities were calculated at the scale of the barn, because i) males never sired extra-pair young outside their own barn, and ii) within the barns extra-pair males often sired young in nests situated a long way from their own nest. The female fertile period was assumed to last from 6 days before clutch initiation until the day the penultimate egg was laid [41]. Because testosterone concentrations of four males were measured in both years, we entered male as a random factor. By entering barn as a random factor, we accounted for the non-independence of males breeding in the same barn. Model selection was based on backward elimination of non-significant terms. Reported values are as in the step prior to elimination from the model or as in the final model.

In male North American barn swallows, the length of the outermost tail feathers and the intensity of the ventral plumage coloration have been shown to affect within and extra-pair fertilization success (e.g. [19], [21]). We therefore included these variables in all analyses of the relationship between male elevated testosterone concentrations and fertilization success. We investigated this relationship from three different angles: we tested whether there was a relationship between male elevated testosterone concentrations and (i) cuckoldry rate, (ii) extra-pair fertilization success, and (iii) total fertilization success. All analyses were performed using multilevel GLMMs in MlwiN 2.0 [40]. As none of the interactions between the independent variables (elevated testosterone, tail length and plumage coloration) and year were significant (all *p*>0.34), data from both years were lumped. By entering barn as a random factor, we accounted for the non- independence of males breeding in the same barn, and by entering male identity as a random factor we accounted for having included males more than once (i.e. males having two nests in one year or breeding in our study area in both years). Cuckoldry rate, i.e. the proportion of EPY in a male's own brood, was analyzed using a binomial error distribution with a logit link function. By entering the number of offspring in a brood as the denominator we accounted for the variation in brood size (one to six nestlings). Because extra-pair fertilization success (the total number of EPY gained in the study population in the entire breeding season) was highly skewed towards males that did not gain EPY, this variable was analyzed in two steps. First, extra-pair fertilization success was analyzed using a binomial error distribution (yes/no EPY) with a logit link function. The denominator was set to 1. Second, extra-pair fertilization success of only those males that did gain EPY was analyzed using a normal error distribution. Total fertilization success, i.e. the total number of offspring produced in the entire breeding season, was analyzed using a normal error distribution.

Because elevated testosterone concentration was significantly correlated with the PC1 of colour (Pearson's r = 0.35, *p* = 0.015 and n = 48), we ran two analyses: one including the variables ‘testosterone concentration’ and ‘tail length’, and one including ‘plumage colouration’ and ‘tail length’. Model selection was based on backward elimination of non-significant terms. Reported values and sample sizes are as in the step prior to elimination from the model or as in the final model. Sample sizes vary because for two males we did not measure testosterone and for one male we could not measure tail length. Probabilities are two-tailed in all tests.

Most of the males that we sampled for elevated testosterone concentrations during their first nests produced a second brood. However, we did not re-trap and blood-sample males around their second broods. Because intra-individual variation in elevated testosterone concentrations may exist between first and second broods, we repeated all analyses that had elevated testosterone concentration as an explanatory variable, this time using only the paternity data of first broods. Results from these analyses did not differ in any significant way from the analyses that included all data and are reported in Appendix S1. Since inclusion of paternity data of second nests did not affect the outcome of the analyses, we used fertilization data from both first and second nests in the figures on fertilization success.

## Results

### Extrinsic factors and timing of sampling

**Figure 1:**
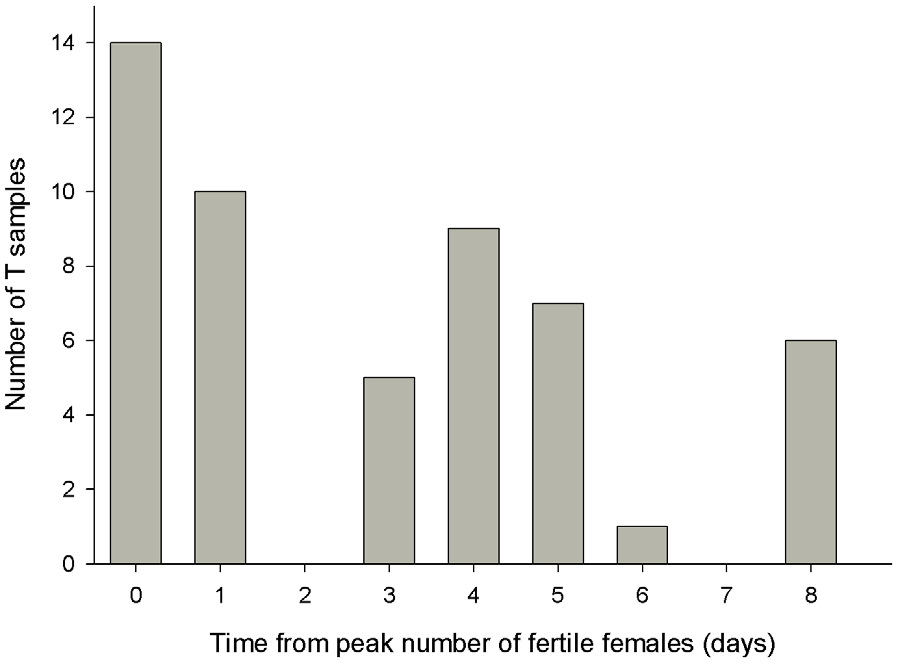
Frequency distribution of the minimum number of days between the peak number of fertile females and testosterone (T) sampling. For each male, the peak number of fertile females was defined as the day, or days, on which the largest number of females was fertile in the barn in which the male nested.

Variation in male elevated testosterone concentration was not related to differences in extrinsic factors at the time of blood-sampling (Table 2). Also, the timing of testosterone sampling was accurate with regard to the period crucial to extra-pair behavior; most testosterone samples were collected at or close to the time when the number of fertile females nesting in the barn of the sampled male was at its peak (Fig. 1).

**Table 2:** Relationships between individual testosterone concentration in male barn swallows and extrinsic factors (n = 51).

Extrinsic factors	β ± SE	χ^2^	df	*P*
Day of capture	−0.033±0.023	2.13	1	0.14
Time of capture	−0.212±8.065	0.01	1	0.97
Ambient temperature at capture	0.006±0.014	0.15	1	0.70
Breeding density	−0.007±0.064	0.01	1	0.91
Opportunities for extra-pair activity	0.03±0.046	0.36	1	0.55

Day of capture was expressed as the number of days before or after the social mate's clutch initiation, breeding density as the number of active nests within 6 meters of the focal bird's nest, and opportunities for extra-pair activity as the number of fertile females present in the barn per male on the day of capture. Ambient tempature ranged from 33.8 to 73.4°F (average 51.8°F), breeding density ranged from 1–10 nests (average 6), and opportunities for extra-pair activity ranged from 2 to 12 fertile females (average 8). Summaries derived from the mixed modelling procedure in MLwiN.

### Fertilization success

#### Cuckoldry rate

Overall in 2009, 55 out of 132 nestlings (42%) resulted from cuckoldry. Extra-pair males sired 30 of 66 nestlings (46%) from first broods and 25 of 66 nestlings (38%) from second broods. Eighteen of 30 broods (60%) contained at least one EPY and seven broods (23%) contained only EPY. Twelve of 16 males (75%) were cuckolded and two males (13%) lost all nestlings to cuckoldry. Overall in 2010, 90 out of 292 nestlings (31%) resulted from cuckoldry. Extra-pair males sired 58 of 172 nestlings (34%) from first broods and 32 of 120 nestlings (27%) from second broods. Thirty-four of 64 broods (53%) contained at least one EPY and six broods (9%) contained only EPY. Thirty-two of 45 males (71%) were cuckolded and two males (4%) lost all nestlings to cuckoldry.

**Figure 2:**
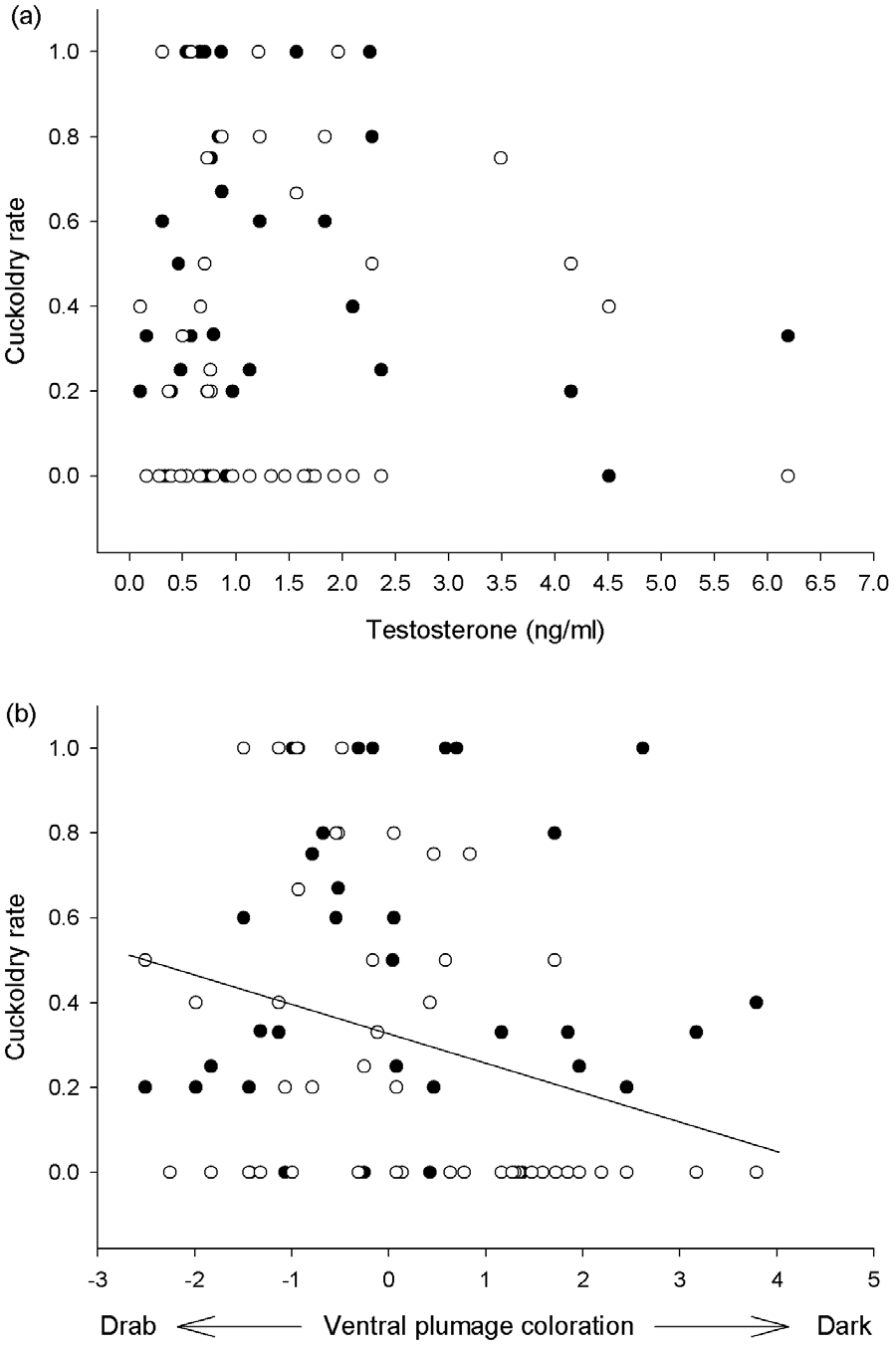
Relationships between cuckoldry rate (i.e. the proportion of extra-pair young in a male's own brood) and (a) male elevated testosterone concentration, and (b) male ventral plumage colour. Filled circles represent first nests and open circles represent second nests. Elevated testosterone concentration did not predict cuckoldry rate. Cuckoldry rate was, however, negatively related to ventral plumage colour. A regression line is presented for the negative relationship between cuckoldry rate and ventral plumage colour.

Elevated testosterone concentration did not predict cuckoldry rate (Table 3; Fig. 2a). There was a near significant negative trend between tail length and cuckoldry rate (Table 3). Cuckoldry rate showed a significant negative relationship with ventral plumage colour (Table 3; Fig. 2b), meaning that dark males lost fewer young to extra-pair paternity than drab males.

**Table 3:** Relationships between phenotype and fertilization success in male barn swallows.

Variables	Cuckoldry rate				EPF Binomial			EPF Normal			Total fertilization		
	β ± SE	χ^2^	*P*	n	χ^2^	*P*	n	χ^2^	*P*	n	χ^2^	*P*	n
Testosterone	−0.06±0.16	0.15	0.70	89	0.47	0.49	51	0.75	0.39	22	0.01	0.94	50
Tail length	−0.07±0.04	3.81	0.051	92	0.40	0.53	50	0.14	0.71	22	0.91	0.34	52
Colour	−0.29±0.12	5.71	0.017	92	0.76	0.76	53	0.63	0.43	22	0.28	0.59	52
Tail length	−0.06±0.04	1.96	0.16	91	0.34	0.56	52	0.12	0.73	22	0.09	0.76	51

Phenotypic variables were: testosterone concentration, ventral plumage colouration and length of the longest tail feather. Because testosterone concentration was correlated with plumage colour, the results of two seperate analyses are presented: one including the variables ‘testosterone concentration’ and ‘tail length’, and one including ‘plumage colouration’ and ‘tail length’. Fertilization success was split into i) cuckoldry rate, i.e. the proportion of extra-pair young in a male's own brood, ii) extra-pair fertilization success (EPF), and iii) total fertilization success, i.e. the total number of offspring produced in the entire breeding season. Extra-pair fertilization success (the total number of EPY gained in the study population in the entire breeding season) was analyzed in two steps; first using a binomial error distribution, and second using a normal error distribution, including only those males that gained extra-pair young (see Methods for rationale). The variance of the random factor ‘Barn’ was not significant in any model (all p>0.05). The variance of the random factor ‘Male’ was significant in all models (all p<0.001). Summaries derived from the mixed modelling procedure in MLwiN. All df = 1.

#### Extra-pair fertilization success

In 2009 we assigned extra-pair males to 37 of 55 EPY (67%). These 37 EPY were sired by six different males, of which two gained as many as 13 and 14 EPY respectively. Two males each gained four EPY and two males each gained one EPY. In 2010, we assigned extra-pair males to 62 of 90 EPY (69%). These 62 EPY were sired by 20 different males, of which two gained as many as 8 and 7 EPY respectively. Two males each gained five EPY, four males gained four EPY, three males gained three EPY, three males gained two EPY, and six males gained one EPY. EPY were always sired by a male that bred in the same barn as the cuckolded male.

When analyzing extra-pair success as a binomial event, elevated testosterone concentration was not related to extra-pair fertilization success, nor was ventral plumage colour or tail length (Table 3). Similarly, when the number of EPY was analyzed as a continuous variable, thus excluding males that did not gain EPY, elevated testosterone concentration was not related to extra-pair fertilization, nor was ventral plumage colour or tail length (Table 3).

#### Total fertilization success

In 2009, the total number of offspring that males sired in the entire breeding season varied from 0 to 21 (mean ± SE = 7.2±1.6). In 2010, the total number of offspring that males sired in the entire breeding season varied from 0 to 16 (mean ± SE = 6.5±0.53).

**Figure 3:**
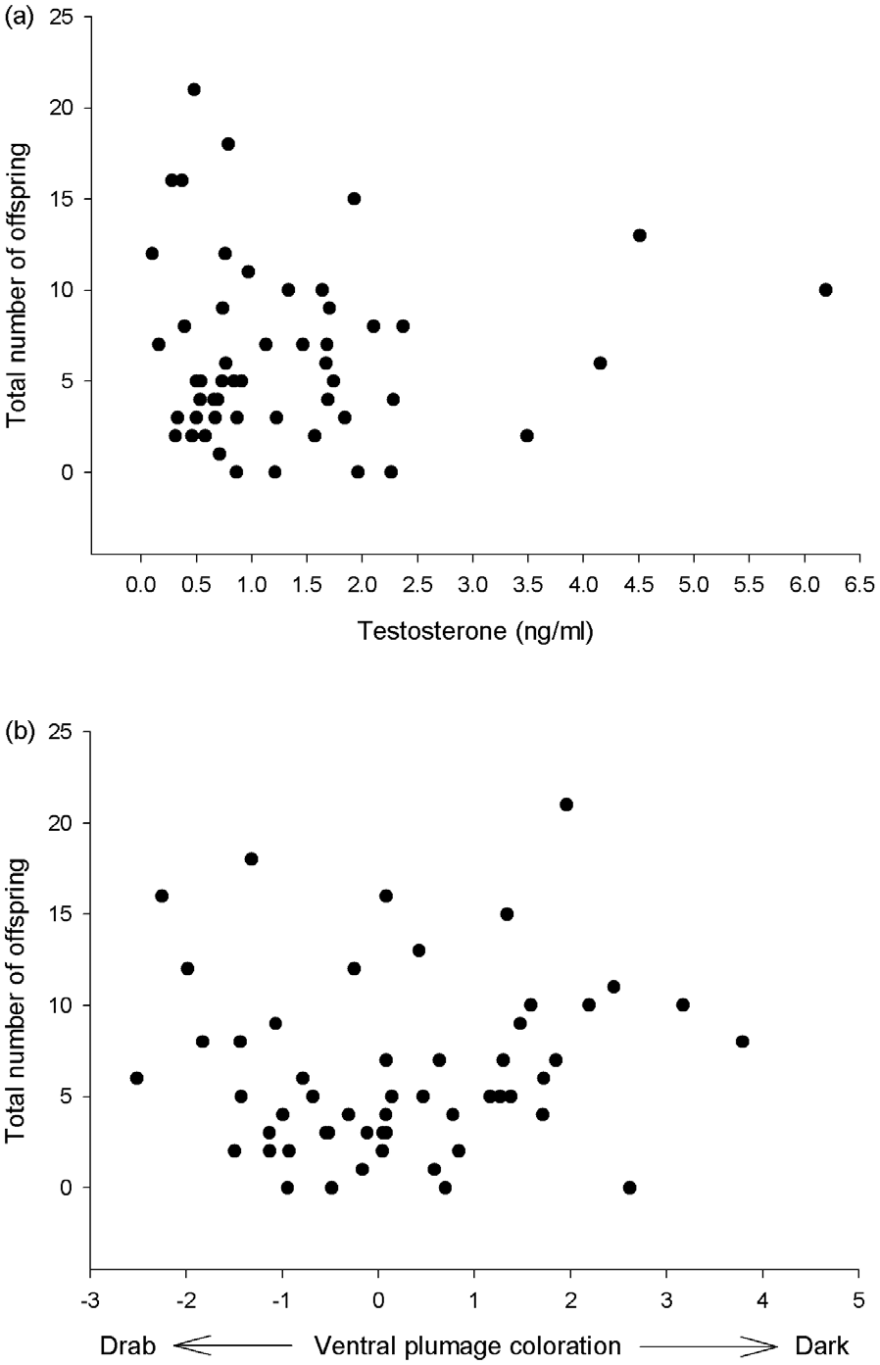
Relationships between total number of offspring produced in a breeding season and (a) male elevated testosterone concentration, and (b) male ventral plumage colour. Elevated testosterone concentration was not correlated with total number of offspring produced, nor was ventral plumage colour.

Elevated testosterone concentration was not correlated with total number of offspring sired (Table 2; Fig. 3a), nor was ventral plumage colour (Table 2; Fig. 3b) or tail length (Table 2).

## Discussion

Male elevated testosterone concentration was not related to cuckoldry rate, extra-pair fertilization success or total fertilization success. Variation in male testosterone concentration could not be explained by differences in extrinsic factors (day, time, ambient temperature, breeding density and opportunities for extra-pair activity) at the time of sampling. Furthermore, most testosterone samples were collected at, or close to the time when the number of fertile females in the barn of the sampled male was at its maximum, i.e. during a time period critical to extra-pair mating success. This shows that the lack of a relationship between testosterone and fertilization success was not due to temporal variation in, and timing of, sampling. Our finding that elevated testosterone concentration was significantly positively correlated with ventral plumage colour also indicates that the variation in elevated testosterone concentration caused by temporal variation in sampling was not substantial. Furthermore, our negative results regarding extra-pair fertilization success do not appear to result from lack of power of our study. For example, our statistical model with the explanatory variables ‘testosterone’ and ‘tail length’ could have detected a medium effect size (f^2^ = 0.20) with a power of 0.8 (post-hoc sensitivity analyses in G*Power 3.1.2 [42]). The lack of a relationship between testosterone and fertilization success indicates that females do not preferentially mate with males that have high elevated testosterone concentrations and suggests that, at least in North American barn swallows, male elevated testosterone concentration does not play a significant direct role in sexual selection. A note of caution should be placed at our results of extra-pair paternity gains. Although males appeared to gain extra-pair paternity close to their own nest (all the extra-pair males that we could assign sired their extra-pair young within their own barn and not in one of the other study barns), we may have missed some extra-pair gains of males that sired young outside of our study area.

In contrast to our results, previous experimental studies in other songbirds found that testosterone implants did affect paternity, but in opposite direction; paternity losses in blue tits [13] and paternity gains in dark-eyed juncos [12]. It would be interesting to investigate whether in these species (extra-pair) fertilization success can be explained by natural variation in male elevated testosterone concentration, especially since the effect of exogenous testosterone on cuckoldry rate was opposite in the two species. Similarly, collecting natural testosterone data may provide insight regarding the positive relationships found between exogenous testosterone and factors that influence reproductive success, such as male attractiveness [10], territory size [43] and vigilance behaviour [9].

Variation in male elevated testosterone concentration may contribute to sexual selection through other routes than extra-pair paternity. A positive correlation between male elevated testosterone concentration and copulation or fledgling success may, at least in part, result from males with higher testosterone being able to secure higher-quality territories (red-winged blackbirds [15]; tawny owls (*Strix aluco*) [39]), occupy the superior positions in a lek (black grouse [2]), or build higher-quality bowers (satin bowerbirds [14]). Testosterone implant experiments may elucidate whether the above mechanisms are truly a cause of the observed relationship between testosterone and reproductive success. Testosterone manipulations could further resolve whether in these systems male elevated testosterone concentration does indeed play a role in sexual selection.

In a previous study, experimentally darkened barn swallow males, but not control males, had reduced cuckoldry rates after manipulation of the ventral plumage [22]. In accordance, our observational study revealed that ventral plumage coloration had a significant effect on cuckoldry rate; the proportion of EPY was smaller in nests of dark males than in nests of drab males. In agreement with another study on barn swallows [44], we also found that naturally dark males had higher testosterone concentrations than drab males. Male testosterone concentration, however, was not correlated with cuckoldry rate. Moreover, naturally dark males did not gain more paternity through extra-pair fertilizations than drab males, which suggests that ventral plumage colour does not reflect male genetic quality per se. We here propose a potential explanation that takes all these findings into account. Possibly plumage colour co-varies with a male behavioural trait that reduces the chance of cuckoldry. Aggressiveness could be such a trait. Red and orange colouration are often associated with dominant and aggressive behaviour [45], [46]. Perhaps a dark rusty brown coloration of the ventral feathers also makes a male barn swallow more aggressive. In support of this idea, a literature review indeed revealed that darker wild vertebrates are more aggressive and sexually active than lighter individuals [47]. Consequently, dark males may be more effective at guarding their females and are therefore less often cuckolded than less aggressive drab males. If plumage colour does indeed co-vary with aggressiveness, this could also explain why naturally dark male barn swallows have higher testosterone concentrations than drab males ([44], this study), and why testosterone concentrations rapidly increased in experimentally darkened male barn swallows [44]. The lack of a relationship between testosterone concentration and cuckoldry rate makes it more plausible that, in the current scenario, aggressiveness affected testosterone concentration and not vice versa. A positive relationship between plumage colouration and energetically demanding aggressive behaviour could further explain why, within one week, experimentally darkened male barn swallows lost body mass while control males did not [44]. Due to such energetic costs, being more aggressive may not increase overall reproductive success, and ventral plumage colour may thus not reflect male genetic quality. Additional research is clearly needed to clarify if indeed dark male barn swallows are more aggressive and guard their mates more effectively than drab males.

Our results on the relationship between tail length and cuckoldry rate were ambivalent. When analysed in a model together with the variable ‘testosterone concentration’, tail length showed a near significant negative trend with cuckoldry rate (P = 0.051), suggesting that males with longer tails are less often cuckolded. This trend, however, was absent when tail length was analysed together with ventral plumage colouration. In general therefore our results agree with previous studies done in a New York population, which concluded that tail length is not a sexually selected trait in North American barn swallows [20], [21]. Is has to be noted though that studies on a population in Ontario found that tail length was negatively related with date of breeding [48], or positively related with extra-pair fertilization success [19], suggesting that tail length is a sexual signal in that population.

Our study is the first to relate natural male elevated testosterone concentration to within and extra-pair fertilization success. We found that in North American barn swallows natural variation in male elevated testosterone concentrations is not associated with social and extra-pair female mate choice. Therefore, although exogenous testosterone may enhance behaviours important for mate choice [8]–[10], it is uncertain whether natural male elevated testosterone concentration plays a significant direct role in female mate choice and sexual selection in socially monogamous birds.

## Supporting Information

Appendix S1 Relationships between phenotype (testosterone concentration and length of the longest tail feather) and fertilization success of male barn swallows calculated from first nests only (most pairs were double brooded). Fertilization success was split into i) cuckoldry rate, i.e. the proportion of extra-pair young in a male's own first brood, ii) extra-pair fertilization success in first nests (EPF), and iii) total fertilization success, i.e. the total number of offspring produced in first nests. Extra-pair fertilization success was analyzed in two steps; first using a binomial error distribution, and second using a normal error distribution, including only those males that gained extra-pair young (see Methods for rationale).
(DOC)
