# Supplementary material for: Testosterone, Plumage Colouration and Extra-Pair Paternity in Male North-American Barn Swallows
Source: PLoS One. 2011 Aug 10;6(8):e23288. doi: 10.1371/journal.pone.0023288 (PMC3154291; doi:10.1371/journal.pone.0023288)
Supplement: Appendix S1 — Relationships between phenotype (testosterone concentration and length of the longest tail feather) and fertilization success of male barn swallows calculated from first nests only (most pairs were double brooded). Fertilization success was split into i) cuckoldry rate, i.e. the proportion of extra-pair young in a male's own first brood, ii) extra-pair fertilization success in first nests (EPF), and iii) total fertilization success, i.e. the total number of offspring produced in first nests. Extra-pair fertilization success was analyzed in two steps; first using a binomial error distribution, and second using a normal error distribution, including only those males that gained extra-pair young (see Methods for rationale). (DOC) [file pone.0023288.s001.doc]

Appendix S1

Relationships between phenotype (testosterone concentration and length of the longest tail feather) and fertilization success of male barn swallows calculated from first nests only (most pairs were double brooded). Fertilization success was split into i) cuckoldry rate, i.e. the proportion of extra-pair young in a male’s own first brood, ii) extra-pair fertilization success in first nests (EPF), and iii) total fertilization success, i.e. the total number of offspring produced in first nests. Extra-pair fertilization success was analyzed in two steps; first using a binomial error distribution, and second using a normal error distribution, including only those males that gained extra-pair young (see Methods for rationale).

| Variables | Cuckoldry rate | | | | EPF Binomial | | | EPF Normal | | | Total fertilization | | |
| --- | --- | --- | --- | --- | --- | --- | --- | --- | --- | --- | --- | --- | --- |
|  | β ± SE | χ^2^ | *P* | n | χ^2^ | *P* | n | χ^2^ | *P* | n | χ^2^ | *P* | n |
| Testosterone | -0.03 ± 0.14 | 0.04 | 0.84 | 49 | 0.05 | 0.82 | 33 | 0.32 | 0.57 | 16 | 0.01 | 0.92 | 33 |
| Tail length | -0.04 ± 0.03 | 2.02 | 0.16 | 50 | 0.02 | 0.89 | 35 | 1.26 | 0.26 | 16 | 0.01 | 0.92 | 35 |

Summaries derived from the mixed modelling procedure in MLwiN. All df =1.
